# Predictability of refraction following immediate sequential bilateral cataract surgery (ISBCS) performed under general anaesthesia

**DOI:** 10.1186/s40662-015-0023-5

**Published:** 2015-08-20

**Authors:** Ivo Guber, Laurent Rémont, Ciara Bergin

**Affiliations:** Jules Gonin Eye Hospital, Foundation Asile des Aveugles, University of Lausanne, Avenue de France 15, 1000 Lausanne 7, Vaud Switzerland; Department of Ophthalmology, Kantonsspital Winterthur, Zürich, Switzerland; Department of Ophthalmology, CHU of Liège, University of Liège, Liège, Belgium

**Keywords:** Cataract, Cataract surgery, Immediate sequential bilateral cataract surgery

## Abstract

**Background:**

To evaluate the predictability of refraction following immediate sequential bilateral cataract surgery (ISBCS) performed under general anaesthesia.

**Methods:**

This is a retrospective review of all ISBCS performed at Kantonsspital Winterthur, Switzerland, between April 2000 and September 2013. The case notes of 250 patients were reviewed. Patients having full refraction reported (110 patients/220 eyes) were included. 210 (95 %) eyes had a straight forward phacoemulsification with posterior chamber intraocular lens implantation, seven eyes had a planned extracapsular cataract extraction (ECCE); three eyes had an intracapsular cataract extraction.

**Results:**

Both eyes of 110 patients (64 women, 46 men) with a mean age of 79.0 years, standard deviation (SD) ±11.4 (range 26 to 97 years) were included. Median preoperative best corrected visual acuity (BCVA) was 0.5 LogMAR in the first eye, the interquartile range (IQR) was [0.4, 1.2]; 0.7 LogMAR in the second eye with IQR [0.4, 1.8]. At one month, the median BCVA was 0.2 LogMAR, IQR [0.1, 0.3] in the first eye, median BCVA was 0.1 LogMAR and IQR [0.0, 0.5] in the second eye. There were 3 eyes (3 %) that lost 3 lines or more in BCVA at one month (control vs. pre-operatively). In all three cases, poor visual acuity had been recorded pre-operatively (>1 LogMAR). Achieved refraction was within ±1.0 D of the target in 83 % of eyes. There were only 5 % (*n* = 6) of cases where if delayed sequential bilateral extraction had been performed could potentially intraocular lens (IOL) choice have been adjusted, in four of these cases, target refraction was within ±1.0 D in the second eye.

**Conclusions:**

ISBCS performed under general anaesthesia achieves target refraction in 83 % of eyes after consideration of complications, ocular co-morbidities and systemic restrictions. In the majority of cases where IOL power calculation could be considered, the achieved refraction of the second surgical eye was within ±1.0 D of intended refraction. This undermines the utility of IOL power adjustments in the second surgical eye.

## Background

While refractive bilateral surgery is nowadays standard practice, the widespread implementation of immediate sequential bilateral cataract surgery (ISBCS) has remained a subject of controversy [1]. A survey of the members of the American Society of Cataract and Refractive Surgeons in 2012 revealed that less than 1 % of surgeons routinely perform ISBCS [2]. There is unfounded fear of intraocular infection associated with ISBCS [3–5], which continues to be cited as the primary reason why this procedure has not been implemented as standard practice. To a lesser extent, reluctance is due to the occasional significant intraocular lens (IOL) power calculation errors in the first eye that can potentially be refined and thereby prevented in the second eye [6].

On the other hand, there are important considerations for the patient in terms of cost and for healthcare services due to the resources required—ISBCS approximately halves the number of required post-operative appointments. In particular, this eases the demand on support infrastructure for mobility restricted patients [7, 8]. With an aging population these considerations will be of increased importance particularly in an era where healthcare is obliged to focus on cost efficiency. Due to the rapid visual rehabilitation and obvious practicalities for elderly patients, some surgeons will perform it on request in the absence of contraindications [9]. Therefore ISBCS is growing in popularity worldwide; some regions such as the Canary Islands have adopted ISBCS as a standard procedure, now 80 % of all cataract surgeries are performed in this way [10].

The very low risk of bilateral complications following ISBCS has been well documented, and will in time erode resistance to the widespread adoption of ISBCS in this respect. This article focuses on the secondary perceived impediment i.e., the possible adjustment of IOL power in the second surgical eye. Therefore, the aim of this study was to calculate the percentage of eyes where adjustments might have proved beneficial in terms of the visual outcome, refractive error and difference from target refraction for the first and second surgical eye.

## Methods

The retrospective study followed the tenets of the Declaration of Helsinki and was reviewed by the internal ethics committee of Kantonsspital of Winterthur, the Canton of Zurich, Switzerland.

### Patients

Patient charts of all consecutive ISBCS performed at the Kantonsspital of Winterthur, Switzerland, performed between April 2000 and September 2013 were reviewed. There were 250 patients in total, only those with refraction available pre- and post-operatively were included in this study (*n* = 110). In general, indication for ISBCS was defined as the need for bilateral cataract surgery (reduced visual acuity with evidence of lens opacity as observed at the slit lamp) and where surgery under local anaesthesia was not feasible due to general health restrictions.

### Calculating IOL power

Biometry was performed with the IOL Master (Carl-Zeiss AG, Oberkochen, Germany). However, in cases of high lens opacity, patients had axial length measured with an A-scan ultrasound (Ocuscan, Alcon, Hünenberg, Switzerland). In five cases, where patients were unable to cooperate, the biometry measurements were completed under general anaesthesia (e.g. Trisomy 21, Parkinson’s disease). Where biometry was completed successfully with the IOL master, the Haigis formula was used to calculate IOL power, elsewhere, the SRK II/SRK T formula was used.

### Surgical procedure

A total of 210 (95 %) eyes of 109 patients had a straight forward bimanual phacoemulsification with posterior chamber intraocular lens implantation. A sclero-corneal tunnel was created in all but one patient where a clear cornea incision was made in both eyes. Seven (3 %) eyes of six patients had a planned extracapsular cataract extraction (ECCE), three (1 %) eyes of three patients had an intracapsular cataract extraction (ICCE) combined with an anterior vitrectomy and prepupillary implantation of an Artisan iris-claw lens (Ophtec B.V., Groningen, Netherlands). Details on IOL implants used are provided in Table 1. All surgical interventions were performed under general anaesthesia. Mean duration of surgery was 52.7 min, standard deviation (SD) ±15.4 (range 33-119). The surgeries of the first and second eyes were performed under complete aseptic separation. In summary, the methods used comply with the general principles of ISBCS [11, 12]: Two different instrument sets were used for surgery and the irrigation fluid was changed. In general, different LOT-Numbers were not used for the left and right eye. Intracameral cefuroxime 0.5 ml (1 mg/0.1 mL) for prophylaxis of endophthalmitis was injected at the end of the surgery. Before surgery of the second eye, the surgeon and nurse undergo sterile routines after independent preparation of the second eye’s operative field. Patients with known α-blocker medication (e.g. Tamsulosin®, Pradif®) were treated with topical Atropine 1 % sid for 5 days before surgery. Blepharitis was treated preoperatively with Tobradex® eyedrops (tobramycin 1 mg/dexamethasone 3 mg) qid for 5 days. If an intraoperative complication occurred in the first surgical eye, the second surgery was postponed.

**Table 1:** Pre and post-operative visual outcomes

Preoperatively		First eye	Second eye
VA (LogMAR)		0.52 (0.39, 1.15)	0.40 (0.15, 0.70)
ACD (mm)		2.97 ± 0.46	2.98 ± 0.47
AL (mm)		23.5 ± 1.6	23.6 ± 1.75
SE (D)		−1.29 ± 4.79	−0.93 ± 4.59
Sphere (D)		−0.72 ± 4.77	−0.34 ± 4.51
Cylinder (D)		−1.30 ± 1.30	−1.27 ± 1.25
Target SE (D)		−1.00 ± 1.13	−0.78 ± 1.40
IOL used			
Plate	Zeiss Asphina CT409MP	33	31
	Acritec	22	22
Iris fixated	Ophtec Artisan	2	2
One piece	Acrysof SN60WF	2	2
	Zeiss CT37A	13	14
	HOYA 251	21	21
	HOYA YA65BB	5	6
	Ophtec PC292Y (PMMA)	3	4
	Polylens Y11P	6	5
	Tecnis PCB00	2	2
Post-operatively		First eye	Second eye
VA (LogMAR)		0.22 (0.10, 0.35)	0.13 (0.01, 0.52)
SE (D)		−1.00 ± 1.13	−0.99 ± 1.40
Sphere (D)		−0.36 ± 1.09	−0.34 ± 1.39
Cylinder (D)		−1.40 ± 1.11	−1.41 ± 1.34
Difference between target and achieved (D)		0.60	0.69

*SE*: spherical equivalence, *VA*: visual acuity, *ACD*: anterior chamber depth, *AL*: axial length

### Postoperative regimen

The postoperative regimen included Tobradex® (tobramycin 1 mg/dexamethasone 3 mg) eye drops qid. The eye drops were tapered over four weeks. Postoperative controls were one day, one week and one month postoperatively. This is a retrospective review of patient case notes. Patients operated under general anaesthesia normally have the day one follow-up visit performed at the hospital. In Switzerland, the primary eye care providers are ophthalmologists rather than optometrists, therefore, week 1 and month 1 follow-up visits were more often performed by the referring ophthalmologist. In this way, only the more complicated cases and those in close proximity to the hospital continued post-operative follow-up at the hospital. All follow-up visits were performed by ophthalmologists. Details on post-operative complications but not refractive outcomes were available for all patients.

## Results

### Patients’ characteristics

In this retrospective study, 250 patient files were reviewed, of which 110 patients (64 women, 46 men) with a mean age of 79.0 years, SD ± 11.4 (range 26 years to 97 years) had suitable data available and were included. In all but two patients, surgeries were performed under inpatient conditions. To date, in this centre, ISBCS is non-standard, reserved only for those cases where it was not possible to perform straightforward cataract surgery under topical anaesthesia, due to the general health condition of the patient (e.g. reduced mobility, nervous system disorders).

#### Ocular co-morbidities

Twenty-four patients (22 %) had no ocular co-morbidities, which could influence surgery/post-operative refraction. Fifty eyes of 33 patients, 30 % of the patient population had a pseudo-exfoliation syndrome of which 16 eyes of nine patients had developed pseudo-exfoliation (PEX) glaucoma. Twelve eyes of eight patients had another type of glaucoma. There are 63 eyes with two or more ocular co-morbidities. The additional information is given in Table 2

**Table 2:** Baseline characteristics and ocular co-morbidities

Baseline	Mean	SD
Age (years)	79.0 years	±11.4 years
- Distance vision target (0 D, -1 D)	88 (80 %)	
- Near vision target (less than -2 D)	22 (10 %)	
First operated eye R/L	50/60	
Ocular comorbidities	Patients	Eyes
None	24	48
PEX (Glaucoma)	33 (9)	59 (16)
Primary open angle glaucoma	7	11
Angle closure glaucoma	1	1
Dry AMD	30	52
Choroidal neovascularisation	1	1
Diabetic retinopathy	7	14
Epiretinal membrane	2	3
Retinal vein occlusion	6	6
Vitreous haemorrhage	1	1
Previous retinal detachment	1	1
Hemianopsie	2	4
Amblyopia	10	10
Monocular vision	8	8
Corneal scar due to herpes	1	1
Asymptomatic cornea guttata	5	10
Blepharitis	25	50
Chronic anterior uveitis	1	1
Enophthalmia	2	4

*AMD*: age related macular degeneration, *PEX*: pseudo exfoliation

.

#### Systemic co-morbidities

One patient had no systemic co-morbidity, 109 patients had at least one systemic comorbidity, but on average patients had 3.4 co-morbidities (1–7). Twenty-five (23 %) patients were receiving oral anticoagulation, 53 patients (48 %) had heart problems (arrhythmia, heart failure, or cardiovascular disease), 66 patients (60 %) had high blood pressure. Thirty-six (33 %) patients had diabetes mellitus. Twenty-seven (25 %) patients had reduced renal function. Nineteen (17 %) patients had reduced respiratory function. Forty (36 %) patients had reduced mobility (e.g. paraplegia), 21 patients were morbidly obese (body mass index (BMI) > 30). Twenty patients (18 %) had suffered cerebral vascular insult. Fifty-five patients had a history of psychological disorder/impairment. Seven patients had a history of cancer.

### Visual acuity

**Fig. 1:**
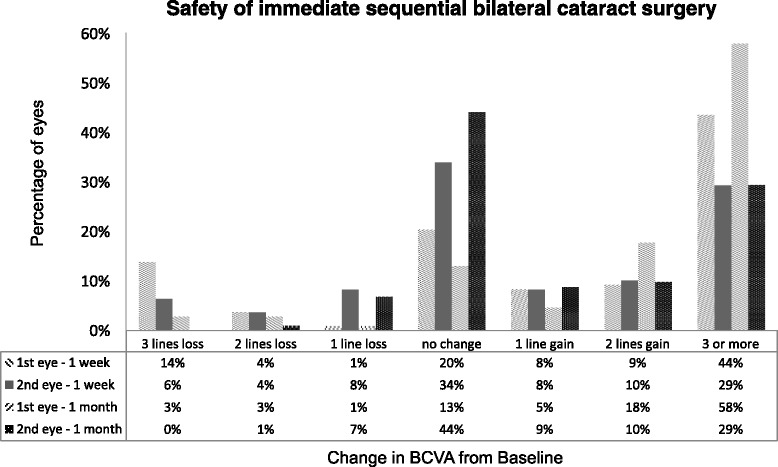
Histogram showing the distribution of the change in BCVA recorded one month after immediate sequential bilateral cataract surgery

Visual acuities were reported according to the recommended guidelines [13]. Median preoperative best corrected visual acuity (BCVA) was 0.5 LogMAR in the first eye, the interquartile range (IQR) was [0.4, 1.2] LogMAR; median BCVA was 0.7 LogMAR with IQR [0.4, 1.8] for the second eye. At week one, the control group’s median BCVA was 0.4 LogMAR, IQR [0.2, 0.5] LogMAR; in the second eye, the median BCVA was 0.2 LogMAR, IQR [0.1, 0.5] LogMAR. At month one, the median BCVA was 0.2 LogMAR, IQR [0.1, 0.3] in the first eye, median BCVA was 0.1 LogMAR IQR [0.0, 0.5]. There were 3 eyes (3 %) that had lost 3 lines or more in BCVA at one month (control vs. pre-operatively). In all three cases, poor visual acuity had been recorded pre-operatively (>1 logMAR). The gain and loss of Snellen lines at one month is shown in Fig. 1. Notably, there were a larger proportion of second surgical eyes that did not gain any Snellen lines post-operatively, this is because the eye with the better visual potential was operated first.

There was no difference observed in myopic eyes (spherical equivalent (SE) < −4.00 D) from the rest of the group (SE > −4.00D) in terms of pre-operative visual acuity, divergence from SE or post-operative visual acuity.

### Formulas used

Eighty-four patients had both eyes measured with optical biometry and the Haigis formula was used to calculate the target SE, this included 88/110 first eyes and 94/110 second eyes measured with optical biometry successfully measured using optical biometry. In the remaining eyes, ultrasound was used with either the SRKII formula (*n* = 20, 13 first eyes/7 second eyes) or SRKT formula (*n* = 18, 9 first eyes/9 second eyes) to calculate the target SE. There was a significant difference in the pre-operative LogMAR BCVA of eyes measured with biometry and ultrasound (0.5 logMAR vs. 1.7 logMAR) and the average difference between target SE and achieved (0.42 D vs 0.73 D).

### Predictability

**Fig. 2:**
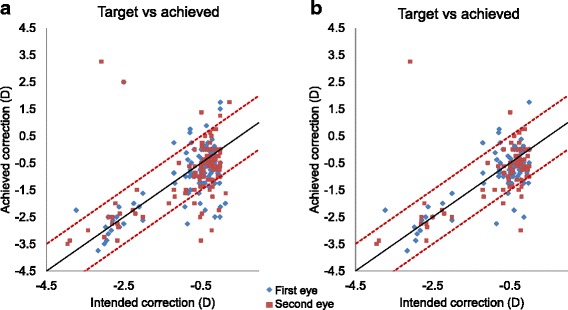
Scatter plot of the achieved correction versus the intended correction one month post immediate sequential bilateral cataract surgery i.e., predictability of refraction post-operatively in (**a**) the full group *n* = 110 and in (**b**) the subgroup with optical biometry available pre-operatively and simple lens exchange surgery (*n* = 78)

In our group, the postoperative refraction was within ±0.5 D of the target in 60 % (*n* = 41) of first surgical eyes and 51 % (*n* = 54) for the second eye. The postoperative refraction was within ±1.0 D in 81 % (*n* = 89) for the first eye and 85 % (*n* = 94) for the second eye. The postoperative refraction was within ±2.0 D in 95 % (*n* = 105) for the first eye and 95 % (*n* = 104) for the second eye (Fig. 2a).

Examining the subgroup of patients with both eyes that had pre-operative optical biometry and simple lens IOL exchange (*n* = 78 patients), the postoperative refraction was within ±0.5 D of the target in 63 % (*n* = 49 patients) of first surgical eyes and 54 % (*n* = 42) for the second eye. The postoperative refraction was within ±1.0 D in 86 % (*n* = 67) for the first eye and 91 % (*n* = 71) for the second eye, and it was within ±2.0 D in 99 % (*n* = 77) for the first eye and 97 % (*n* = 76) for the second eye (Fig. 2b).

Twenty patients had post-operative refraction greater than 1.0 D from target SE in their first operated eye. Two of these eyes had intraoperative complications; in three eyes, biometry could only be achieved under general anaesthesia; one eye developed choroidal neovascularisation postoperatively. Pre-operatively, one eye had choroidal neovascularisation, four had age related macular degeneration (AMD), one had strabismus. Two patients had dementia thus resulting in unreliable refraction results. Therefore, there were 6 patients who based on the visual outcome of the first eye, could have considered adjustments to the IOL power calculation. In 4 of these 6 patients, the refractive outcome of the second eyes was within ±1.0 D.

### Complications

In 98 patients, there were no intraoperative or postoperative complications observed in either eye. In the remaining 12 patients, one or more complications were observed unilaterally, there were no cases of bilateral complications.

#### Intraoperative

In one eye, an accidental sulcus implantation led to a rhexis fixated optic. An intraoperative conversion to ICCE because of total zonula dialysis was performed in three eyes (1 %). One IOL implant broke during insertion, and consequently was replaced intraoperatively. In 18 eyes of ten patients (9 %), an intraoperative floppy-iris syndrome (IFIS) was noted, and in one of these eyes, an iris prolapse occurred intraoperatively requiring a sectorial iridectomy.

#### Postoperative

Postoperative hypertony (IOP > 30 mmHg) was observed in 3 eyes (1 %), ICCE has been performed in two of these eyes. One eye after ECCE had a postoperative wound dehiscence with iris incarceration (due to eye rubbing). One eye (1 %) had a corneal decompensation (preoperatively Fuchs dystrophy had not been observed but known PEX glaucoma patient). Following uncomplicated phacoemulsification, postoperative conversion from a dry AMD to a wet AMD was observed in one eye. One eye had reactivation of herpes keratitis. One eye suffered from a prolonged anterior chamber inflammation (toxic anterior segment syndrome (TASS) suspect).

## Discussion

In most countries, ISBCS is done as the exception rather than the rule, ophthalmic surgeons state that the fear of bilateral sight-threatening complications is the limiting factor, in reality there is a very low risk of bilateral complications [14]. The second and lesser reservation of surgeons when performing ISBCS is the loss of the ability to adjust their IOL calculation for the second eye following the visual outcome of first eye. This study demonstrated the relatively small deviation from target spherical equivalence in the majority of cases of ISBCS, in particular, these results highlighted the small percentage of cases where IOL calculation could be practically employed. These results combined with the good final visual acuity of patients who underwent ISBCS, adds further evidence to support a more general acceptance of ISBCS clinically.

Deviation from target spherical equivalence and postoperative BCVA outcomes reported here agree well with earlier retrospective studies [15, 16]. Johansson et al. reported similar predictability: within 1.0 D was achieved in 71 % of eyes in comparison to the 83 % reported here, any difference may be due to inconsistencies between IOL calculation methodologies (optical vs ultrasound) [16]. Our results were also in line with the results reported from prospective large studies [17, 18], however, it is worth noting that unlike these prospective studies, the group reported here had no eligibility criteria. The results of this article are more representative of the general cataract population, with multiple ocular and systemic co-morbidities. In a subgroup of these patients, where optical biometry was available bilaterally pre-operatively and where simple crystalline lens-IOL exchange was performed, we observed that predictability increased, surpassing results reported in the other studies [15].

Olsen et al. suggested a correction of the IOL calculation of left eye based on the outcomes of the right eye that may be beneficial. This correction could theoretically increase the predictability in the second eye (increasing the number of eyes within ±1.0 D) [19]. In our study group, only 20 patients had a refraction that was greater than 1.0 D from target SE in their first operated eye. Examining these patients in detail, we observed that many patients had additional considerations that would have inhibited the adjustment of IOL power calculations. Finally, there were only 6 patients who based on the visual outcome of the first eye, could have considered adjustment to the IOL power calculation. In four of these six patients, the second eye achieved a refraction that was within ±1.0 D of the target (with no adjustment based on achieved refraction of the first eye), therefore there were two eyes that could have potentially benefitted from such an adjustment, and four that potentially could have resulted in a worse result due to any such adjustment. This suggests that IOL calculation adjustment based on the surgical outcome of the first eye is not advisable as a rule.

As a retrospective study, the available data is not directly comparable to those reported in prospective studies. For example, the preoperative measurements, the surgical techniques/surgeon, and the formulas chosen to calculate the implanted IOL are not uniform; these inconsistencies will inevitably influence the postoperative refraction. Moreover, there was no pre-screening of patients for inclusion or exclusion criteria, therefore the patients included here are likely more complex. On the other hand, a group of senile patients with a multitude of ocular and general comorbidities is more typical of general clinical case mix. Lastly, since the refraction was measured in the clinic, it can be assumed that a proportion of visual acuity measures were sub-optimal. These factors will impact the reproducibility of our results.

There is a potential bias in the patient selection, as only 110 patients had pre- and post-operative refraction available from the full consecutive series of 250 ISBCS surgeries. Unlike other countries, the operating policy in Switzerland is to return the patient immediately to the referring ophthalmologist, with only the more complicated cases and those in close proximity to the hospital returning to the tertiary centre for post-operative follow-up. This could potentially have influenced our results negatively, for example here we observed three conversions to ICCE intraoperatively, however in all the 250 patients notes reviewed, there were no additional cases of conversion to ICCE.

The formulas used here were the Haigis, SRKT and SRKII. It is likely that the universal choice (where available) of the Haigis formula for IOL power calculation, was suboptimal for longer or shorter eyes. In this study, there was significant difference in the improvement from pre- to post-operative BCVA, and it achieved refraction between the eyes calculated with Haigis and SRK formulae. However, this was likely due to the more complicated cases estimated with the ultrasound rather than any unsuitability of formula choice. There was no significant difference in the divergence from target SE with myopia, indicating that this was not a highly significant contributing factor.

## Conclusion

This retrospective study demonstrates that ISBCS is a safe surgical option in terms of refraction. The good predictability of SE observed here, indicates that only in a small proportion of eyes could IOL calculation be beneficial between surgery of the first and second eye. This suggests that there is little merit to delaying the second surgery with this aim.

## References

[1] Jhanji V, Sharma N, Prakash G, Titiyal JS (2007). Simultaneous bilateral cataract surgery in developing countries. J Cataract Refract Surg..

[2] Analyze, Inc. 2012 Survey of US ASCRS Members. Available at: http://www.analeyz.com/NEWAnaleyz%20ASCRS%202012.htm. Accessed December 3, 2013.

[3] Arshinoff S (2008). Bilateral endophthalmitis after simultaneous bilateral cataract surgery. J Cataract Refract Surg..

[4] Guber I, Bergin C, Stürmer J (2014). More than 10 years of experience with immediate sequential bilateral cataract extraction (ISBCE)—a retrospective study. Open J Ophthalmol.

[5] Li O, Kapetanakis V, Claoué C (2014). Simultaneous bilateral endophthalmitis after immediate sequential bilateral cataract surgery: what's the risk of functional blindness?. Am J Ophthalmol..

[6] Henderson BA, Schneider J (2012). Same-day cataract surgery should not be the standard of care for patients with bilateral visually significant cataract. Surv Ophthalmol..

[7] Leivo T, Sarikkola AU, Uusitalo RJ, Hellstedt T, Ess SL, Kivelä T (2011). Simultaneous bilateral cataract surgery: economic analysis; Helsinki Simultaneous Bilateral Cataract Surgery Study Report 2. J Cataract Refract Surg..

[8] Arshinoff SA, Chen SH (2006). Simultaneous bilateral cataract surgery: financial differences among nations and jurisdictions. J Cataract Refract Surg..

[9] Arshinoff SA (2012). Same-day cataract surgery should be the standard of care for patients with bilateral visually significant cataract. Surv Ophthalmol..

[10] Government of Spain publication. Seguridad, efectividad y coste efectividad de la cirugía de cataratas bilateral y simultá- nea afrente a la cirugía bilateral de cataratas en dos tiempos. Informes de Evaluación de Tecnologías Sanitarias SESCS Núm. 2006/05. Available at: http://www3.gobiernodecanarias.org/sanidad/scs/content/660372e4-1f33-11e0-964e-f5f3323ccc4d/2006_05.pdf. Accessed November 19, 2013

[11] Arshinoff SA (2006). Need for strict aseptic separation of the 2 procedures in simultaneous bilateral cataract surgery. J Cataract Refract Surg..

[12] International Society of Bilateral Cataract Surgeons. General Principles for Excellence in iSBCS 2009. Available at: www.isbcs.org. Accessed November 19, 2013.

[13] Stulting RD, Dupps WJ, Kohnen T, Mamalis N, Rosen ES, Koch DD (2011). Standardized graphs and terms for refractive surgery results. Cornea.

[14] Arshinoff SA, Bastianelli PA (2011). Incidence of postoperative endophthalmitis after immediate sequential bilateral cataract surgery. J Cataract Refract Surg.

[15] Sarikkola AU, Kontkanen M, Kivelä T, Laatikainen L (2004). Simultaneous bilateral cataract surgery: a retrospective survey. J Cataract Refract Surg..

[16] Johansson B (2004). Resulting refraction after same-day bilateral phacoemulsification. J Cataract Refract Surg.

[17] Serrano-Aguilar P, Ramallo-Fariña Y, Cabrera-Hernández JM, Perez-Silguero D, Perez-Silguero MA, Henríquez-de la Fe F (2012). Immediately sequential versus delayed sequential bilateral cataract surgery: safety and effectiveness. J Cataract Refract Surg.

[18] Sarikkola AU, Uusitalo RJ, Hellstedt T, Ess SL, Leivo T, Kivelä T (2011). Simultaneous bilateral versus sequential bilateral cataract surgery: Helsinki Simultaneous Bilateral Cataract Surgery Study Report 1. J Cataract Refract Surg.

[19] Olsen T (2011). Use of fellow eye data in the calculation of intraocular lens power for the second eye. Ophthalmology..

